# Next-Generation Intensity-Duration-Frequency Curves for Diverse Land across the Continental United States

**DOI:** 10.1038/s41597-023-02680-4

**Published:** 2023-12-04

**Authors:** Hongxiang Yan, Zhuoran Duan, Mark S. Wigmosta, Ning Sun, Ethan D. Gutmann, Bert Kruyt, Jeffrey R. Arnold

**Affiliations:** 1 Earth Systems Science Division, Pacific Northwest National Laboratory, Richland, WA USA; 2 Department of Civil and Environmental Engineering, University of Washington, Seattle, WA USA; 3 National Center for Atmospheric Research, Boulder, CO USA; 4 MITRE Corporation, McLean, VA USA

**Keywords:** Hydrology, Hydrology

## Abstract

The current methods for designing hydrological infrastructure rely on precipitation-based intensity-duration-frequency curves. However, they cannot accurately predict flooding caused by snowmelt or rain-on-snow events, potentially leading to underdesigned infrastructure and property damage. To address these issues, next-generation intensity-duration-frequency (NG-IDF) curves have been developed for the open condition, characterizing water available for runoff from rainfall, snowmelt, and rain-on-snow. However, they lack consideration of land use land cover (LULC) factors, which can significantly affect runoff processes. We address this limitation by expanding open area NG-IDF dataset to include eight vegetated LULCs over the continental United States, including forest (deciduous, evergreen, mixed), shrub, grass, pasture, crop, and wetland. This NG-IDF 2.0 dataset offers a comprehensive analysis of hydrological extreme events and their associated drivers under different LULCs at a continental scale. It will serve as a useful resource for improving standard design practices and aiding in the assessment of infrastructure design risks. Additionally, it provides useful insights into how changes in LULC impact flooding magnitude, mechanisms, timing, and snow water supply.

## Background & Summary

Hydrological extreme events, such as floods and droughts, have a significant impact on human society and the natural environment^1–4^. In the western United States, over 50% of the water supply comes from mountain snowmelt^5–7^. Insufficient winter snowpack can result in water shortages and environmental strains during dry summer months^8–11^, whereas deep snowpack accompanied by warm temperatures and rain can result in rapid melting and consequent rain-on-snow (ROS) flooding. Without appropriate mitigative measures, these floods can cause extensive damage to infrastructure and property^12–14^. An instance of such a flood occurred in Yellowstone National Park in 2022, which forced the park’s closure for the first time in 34 years and may cost more than $1 billion for rebuilding damaged bridges and roads^15^.

At present, there is a lack of a consistent and systematic hydrological design approach for snow-dominated regions of the United States^16–18^. Local design manuals require or recommend the use of precipitation-based intensity-duration-frequency (PREC-IDF) curves, such as the National Oceanic and Atmospheric Administration Atlas 14^19^. However, the PREC-IDF method implicitly assumes that precipitation is in the form of rain and immediately available for the rainfall-runoff process, which can result in significant underestimation of flooding caused by snowmelt or ROS events (i.e., underdesign)^7,20–22^. For instance, the infrastructure in Yellowstone National Park was not constructed to withstand ROS flooding^23^. To address this need, Yan, *et al*.^20^ proposed next-generation IDF (NG-IDF) curves, which enhances the PREC-IDF approach for hydrological design in regions dominated by both rainfall and snow. The NG-IDF curves characterize the water available for runoff (W) from rainfall, snowmelt, and ROS events. Yan, *et al*.^24^ compared extreme events estimated from NG-IDF with those from PREC-IDF, using observed precipitation and snow water equivalent (SWE) data from almost 400 Snowpack Telemetry (SNOTEL) stations across the western United States. They discovered that around 70% of these stations were subject to underdesign when using the PREC-IDF method, leading to underestimations of floods up to 324%. To expand the use of NG-IDF curves from SNOTEL stations to ungauged sites, Sun, *et al*.^25^ employed a validated physics-based hydrological model, the Distributed Hydrology Soil Vegetation Model (DHSVM)^26–28^, to develop NG-IDF curves under open condition across the continental United States (CONUS) at a 1/16° (~6 km) resolution (>200,000 sites).

Despite these advancements, NG-IDF research to date has been applied to open condition without accounting for the influence of land use land cover (LULC) on W such as canopy interception and interactions with snow, etc. For instance, forest canopy can enhance peak SWE levels and prolong the duration of snowpack^29^, consequently leading to an elevation in the occurrence of ROS events^30^. The W response to LULC is specific to each location and influenced by the local climate and vegetation conditions. Furthermore, at a particular location, the W response may vary from year to year based on the prevailing meteorological conditions. For water resources planning under nonstationarity, changes in LULC, such as deforestation, have the potential to increase peak SWE and subsequently summer water supply^29,31,32^. However, they can also contribute to more intense occurrences of flooding^33,34^.

To address this gap and build upon the work of Sun, *et al*.^25^, we have extended the NG-IDF datasets from open condition to include eight vegetated LULCs over the CONUS, namely deciduous forest, evergreen forest, mixed forest, shrub, grass, pasture, crop, and wetland. These LULCs were chosen in alignment with the National Land Cover Database (NLCD)^35^ and National Resource Conservation Service (NRCS) Technical Release 55 (TR-55)^36^. The updated NG-IDF (NG-IDF 2.0) datasets cover more than 200,000 sites across the CONUS with a resolution of approximately 6 km. The NG-IDF 2.0 dataset spans the years 1951–2013 and includes a total of nine LULCs, including eight vegetated LULCs and the open condition. For each LULC, the datasets encompass daily time series data of W and SWE. They provide comprehensive information on hydrological extreme events and their associated hydrometeorological drivers. This information also proves useful in assessing the risk of infrastructure design and examining the impact of changes in LULC on the annual peak SWE, which is an indicator of potential summer water supply.

## Methods

### Water available for runoff modeling

**Fig. 1:**
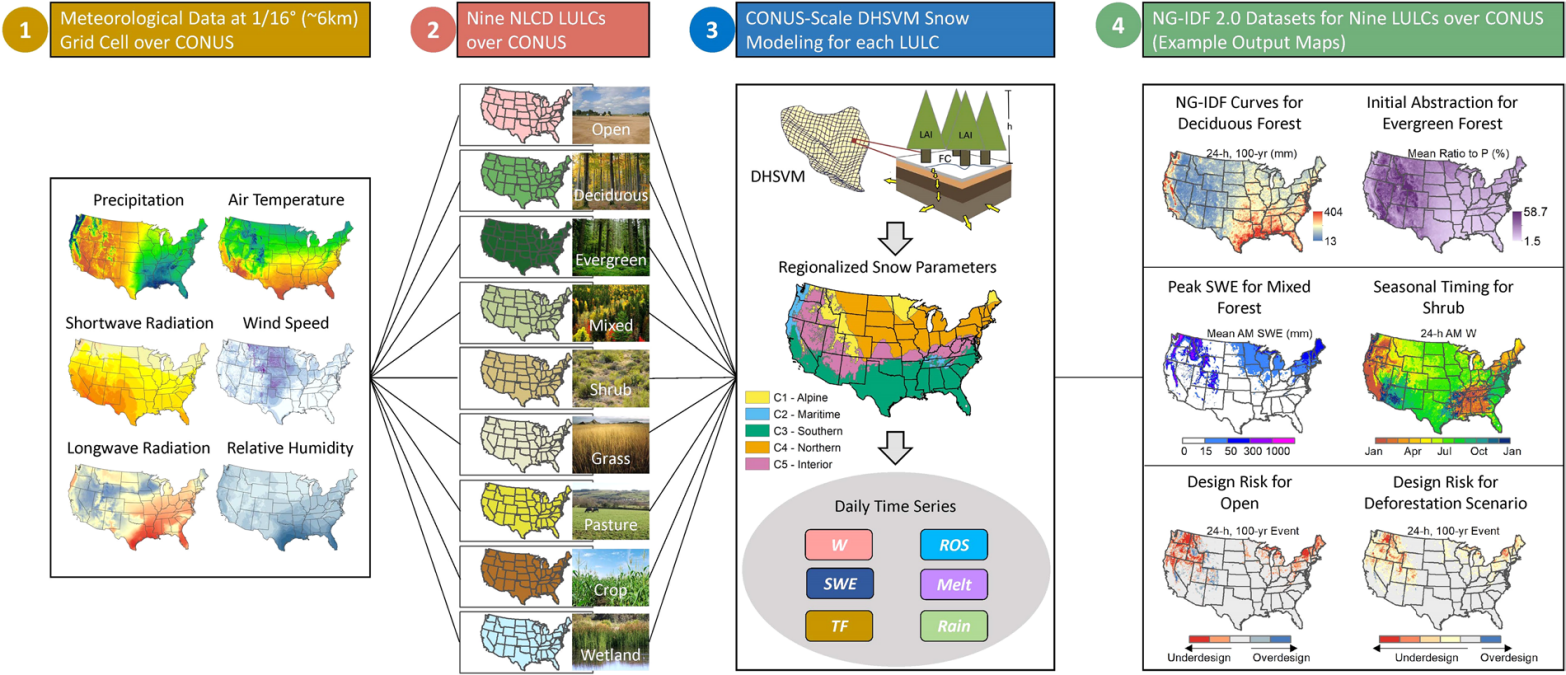
Diagram depicting the methodology used to generate NG-IDF 2.0 datasets for nine different LULCs across the CONUS and their resulting representation.

Figure 1 illustrates the methodology used to create NG-IDF 2.0 datasets for nine different LULCs across the CONUS. In this study, we adopt an approach where we assume a uniform LULC (i.e., 100% canopy fractional coverage) across the entire CONUS area, instead of representing the actual variation of LULC across the landscape. For each specific LULC, we separately estimate the time series of W. This approach is better suited for planning evaluations, by facilitating comparisons across locations. For instance, comparisons of W for the same locations with different LULCs will reveal the effects of LULC change on W (e.g., deforestations vs. afforestation), and cross-location comparisons will yield insights into the climate control on W under different LULC conditions. In this study, DHSVM is utilized to simulate the interaction between rainfall/snowfall and canopy at the point scale. The model incorporates a two-layer canopy model, an overstory canopy snow model, and a two-layer below-canopy energy and mass balance snowpack model. Comprehensive documentation of DHSVM can be found in numerous literature sources^26–28,37^, and as such, we only provide brief model descriptions here.

With the presence of a canopy, throughfall (TF) is generated when canopy interception storage exceeds the maximum interception storage capacity of overstory and understory, respectively. The model first calculates intercepted rainfall and snowfall by overstory if present. Maximum canopy interception of rain and snow is determined as a function of leaf area index (LAI). The water intercepted by the canopy is subject to evapotranspiration (ET). Potential evaporation is first calculated and represents the maximum rate at which water can be removed from the canopy. Water intercepted by the overstory is removed from the wet fraction at the potential rate, while transpiration from the dry fraction is modeled using the Penman-Monteith approach. The understory ET is then calculated as the difference between the potential evaporation and calculated overstory ET (both wet and dry fractions). When a ground snowpack is present, excess snowfall and rainfall after interception combined with the mass release and drip from the overstory will contribute energy and mass to the ground snowpack. The snowpack model is a two-layer snow model with a thin surface layer and a lower pack layer. Energy balance at the snow surface layer is driven by net radiation, sensible and latent heat, and advected heat by rain. The surface layer receives attenuated shortwave radiation below the overstory and direct shortwave radiation in the open. Energy and mass exchange between the surface layer and the pack layer occurs only via the exchange of meltwater. Any liquid water remaining in the pack layer above its liquid water-holding capacity is released into the soil.

When simulating the open area condition without a canopy, W is estimated using mass balance as described by Sun, *et al*.^25^:

1 $${\rm{Open}}\;{\rm{area}}:{\rm{W}}={\rm{P}}-\Delta {\rm{SWE}}+{\rm{S}}$$

where P is precipitation, ∆SWE is the change in ground SWE, and S indicates condensation (positive) or evaporation/sublimation (negative) of the snowpack. Taking into account the canopy impact on runoff, W under the canopy is estimated using:

2 $${\rm{Under}}\;{\rm{canopy}}:{\rm{W}}={\rm{TF}}-\Delta {\rm{SWE}}+{\rm{S}}$$

where TF is the throughfall after canopy rain/snow interception, subsequent evaporation/sublimation, and in the case of snow, melt and mass release through sloughing. TF explicitly quantifies the initial abstraction of vegetation, in contrast to P used for the open condition.

### DHSVM input and parameterization

DHSVM is set up to model runoff processes at the point scale, covering the period from 1950 to 2013 with a 3-hour time resolution. The simulations are carried out on grid cells that correspond to the center of the Livneh 1/16° meteorological grid^38^. DHSVM’s meteorological inputs consist of 3-hourly precipitation, air temperature, wind speed, relative humidity, as well as downward shortwave and longwave radiation. The 3-hourly meteorological forcing data were generated by disaggregating the daily Livneh meteorological data^38^ using the Mountain Microclimate Simulation Model^39^. For ground snow processes, we used the same, validated regionalized snow parameters documented in Sun, *et al*.^25^ for modeling ground snow accumulation and melt (Supplementary Table 1). Specifically, they used the *k*-means clustering technique based on the grid-level winter (November–March) precipitation, air temperature, and wind speed to classify the CONUS into five homogenous regions for snow parameterization: C1-Alpine, C2-Maritime, C3-Southern, C4-Northern, and C5-Interior (Fig. 1).

For a given LULC type, canopy parameters such as monthly LAI and height can be high variably across the CONUS, let alone account for dynamic vegetation and LULC changes due to human activities. To facilitate cross-location comparisons, we use the developed five clusters across the CONUS to represent the spatial variability of canopy parameters, and uniform canopy parameters are employed within each cluster. The cluster-specific canopy parameters represent the average canopy condition over the cluster, determined by averaging the LAI and canopy height measurements obtained from locations within the cluster. Further details about data sources are described in the following paragraph.

A series of datasets are utilized to determine canopy parameters in each cluster. First, the latest 2016 NLCD dataset^35^ and the Landscape Fire and Resource Management Planning Tools Project (LANDFIRE) Existing Vegetation Height (EVH) database^40,41^ are used in tandem to determine the canopy height for each of the eight vegetated LULCs at a 30-m resolution across the CONUS. Second, LAI values are obtained from combining field measurements and remotely sensed products, including the Moderate Resolution Imaging Spectroradiometer (MODIS) Version 6 LAI products MCD15A2H^42^, North American Carbon Program Terrestrial Ecosystem Research and Regional Analysis-Pacific Northwest (NACP TERRA-PNW) Forest Plant Traits^43^, and A Global Database of Field-observed Leaf Area Index in Woody Plant Species (LAI_WOODY_PLANTS_1231)^44^. The NACP TERRA-PNW datasets provide LAI measurements for overstory trees in Oregon and Northern California, while the LAI_WOODY_PLANTS_1231 datasets provide global measurements of overstory and understory LAI from 1,216 locations based on literature sources published between 1932 and 2011. Specifically, we use the 8-day, 500-m MODIS data to characterize the spatial variability and sub-seasonal changes of LAI; field data is chosen over MODIS to derive the maximum LAI values, because MODIS tends to underestimate LAI values at a local scale^45,46^. In line with the NLCD land cover classification, the forest types (deciduous, evergreen, mixed) and wetland land cover categories exhibit both overstory trees and understory vegetation, whereas the shrub, grass, pasture, and crop land cover categories exclusively feature understory vegetation. For example, Table 1 shows the cluster-average parameter values of maximum LAI and height for overstory and understory of deciduous forest, as well as the monthly LAI ratios to the maximum LAI values. Canopy parameter values for the other seven LULCs are presented in Supplementary Tables 2–8.

**Table 1:** Cluster canopy parameters of deciduous forest developed for the CONUS runs.

Canopy Parameter		Cluster				
		C1-Alpine	C2-Maritime	C3-Southern	C4-Northern	C5-Interior
Overstory Max LAI		4.5	5.3	4.9	5.2	5.5
Understory Max LAI		0.5	1.6	1.2	0.7	0.4
Overstory Height (m)		16.1	21.2	19.0	18.9	21.0
Understory Height (m)		0.5	0.9	0.7	0.5	0.4
Monthly LAI Ratio to Max LAI	January	0.10	0.27	0.19	0.22	0.21
	February	0.12	0.32	0.24	0.23	0.25
	March	0.19	0.41	0.33	0.32	0.34
	April	0.26	0.67	0.63	0.43	0.55
	May	0.57	0.99	0.96	0.68	0.80
	June	1.00	0.97	1.00	0.94	0.97
	July	0.91	1.00	0.97	1.00	1.00
	August	0.74	1.00	0.84	0.97	0.95
	September	0.55	0.82	0.70	0.79	0.84
	October	0.23	0.67	0.51	0.49	0.57
	November	0.18	0.35	0.27	0.27	0.28
	December	0.11	0.26	0.19	0.22	0.22

### NG-IDF curves

We aggregated the 3-hourly W time series to create NG-IDF curves for selected durations ranging from 24 to 72 hours due to the absence of diurnal variability in the input precipitation data. For each duration, we determined the water year and calendar year annual maximum (AM) W and followed the NOAA Atlas 14^19^ to fit a generalized extreme value (GEV) distribution to the 1951–2013 AM W datasets based on L-moments statistics^47^, excluding the first year to avoid initial condition uncertainty. We used the same GEV distribution to compare frequency estimates across durations and locations. We tested the stationarity assumption of the AM W data using the nonparametric Mann-Kendall test^48,49^ and provided trends for statistically significant cases at the 95% confidence level. For significant trends, we detrended the AM W time series using Sen’s slope^50^ while maintaining the time series average. In total, we created four NG-IDF curves for each location and LULC, using 1) water year AM W, 2) detrended water year AM W, 3) calendar year AM W, and 4) detrended calendar year AM W for average recurrence interval (ARIs) of 2, 5, 10, 25, 50, 100, and 500 years. A Monte Carlo (MC) simulation method^19,47^ was used to consider sample data uncertainty in frequency analysis. After estimating the parameters of GEV distribution using the L-moments statistics, a total of 1,000 MC synthetic data sets were generated with the same record length. We then fitted GEV distribution to each MC synthetic data set using the L-moments statistics and estimated the associated values of the selected ARIs. Therefore, a total of 1,000 ensemble members were generated to quantify the uncertainties associated with NG-IDF curves. We provided the 90% confidence intervals using the 5% and 95% quantiles of the ensemble members. To provide a reference for comparison, we utilized the same methodology to create PREC-IDF curves with water year AM P and calendar year AM P. All trend and MC analyses were performed using the “trend”^51^ and “lmom”^52^ package in R, an open-source software environment.

### Driving mechanism and seasonality

The driving mechanism of W was identified for each location, duration, and LULC. The classification of the driving mechanism was based on the P/TF and ∆SWE, as per Sun, *et al*.^25^, and included three categories:

1. Rainfall only, which refers to precipitation or throughfall on snow-free ground;
2. Snowmelt only, which refers to decreasing SWE with no concurrent precipitation or throughfall; and
3. ROS, which refers to decreasing SWE with concurrent precipitation or throughfall. To further refine the focus on flood potential, a ROS event was defined as having at least 10 mm of precipitation or throughfall per day falling on a snowpack with at least 10 mm SWE over the selected duration. Additionally, the sum of rain and snowmelt had to contain at least 20% snowmelt^13,25,53,54^.

Precipitation was used for open area while throughfall was used for vegetated LULCs. In addition to the AM W time series, the AM Rain, AM Melt, and AM ROS time series were also provided for each location, and design events were constructed using the same approach as described above. For each location, the driving mechanism that produced the largest design event was identified as the dominant mechanism of hydrological extreme events. The degree of seasonality exhibited by AM W at different durations and LULCs was quantified using circular statistics to calculate the seasonality index (SI) and mean date (MD). While the mathematical details of circular statistics have been extensively covered in previous literature^55–57^, interested readers may refer to Sun, *et al*.^25^ for more information. The SI is a value between 0 and 1, with higher values indicating a greater degree of seasonality, while the MD provides insight into the average timing of AM W events.

## Data Records

The NG-IDF 2.0 datasets covering the CONUS are publicly available in ASCII format through nine Zenodo repositories. These repositories host the datasets for various land cover types, including: (1) Evergreen forest^58^. (2) Deciduous forest^59^. (3) Mixed forest^60^. (4) Grassland^61^. (5) Crop^62^. (6) Open area^63^. (7) Pasture^64^. (8) Shrub^65^. (9) Wetland^66^. All repositories follow identical data structures, and Table 2 presents a summary of the data structures, data files, and variables specifically for the evergreen forest as an illustrative example.

**Table 2:** Description of the NG-IDF 2.0 datasets for evergreen forest.

Main Folder	Naming Convention	Data File Description*
/24-h_time_series/	daily time series from 1950/1/1 to 2013/12/30	
	data_[lat]_[lon] e.g., data_25.15625_−80.71875	**Data Dimension**: 23,375 (R) × 4 (C). **C1**: W; **C2**: P; **C3**: TF; **C4**: SWE, all in mm.
/AM_time_series_[Year]/e.g., /AM_time_series_CY/	AM series with durations of 24 h, 48 h, and 72 h driven by different mechanisms from CY 1950–2013, including melt, rain, ROS, TF, and W. AM SWE series is also included.	
	[duration]_AM_time_series/[mechanism]/data_[lat]_[lon] e.g., 24-h_AM_time_series/W/data_25.15625_−80.71875	**Data Dimension**: 64 (R) × 4 (C) **C1**: Year; **C2**: Month; **C3**: Day; **C4**: AM value in mm.
/d_AM_time_series_[Year]/e.g., /d_AM_time_series_CY/	Detrended AM series with durations of 24 h, 48 h, and 72 h driven by different mechanisms from CY 1951-2013.	
	[duration]_AM_time_series/[mechanism]/data_[lat]_[lon] e.g., 24-h_AM_time_series/W/data_25.15625_−80.71875	**Data Dimension**: 63 (R) × 4 (C) **C1**: Year; **C2**: Month; **C3**: Day; **C4**: AM value in mm.
/IDF_curves_[Year]/e.g., /IDF_curves_CY/	IDF values and their 90% confidence intervals with durations of 24 h, 48 h, and 72 h driven by different mechanisms from 1951-2013, including melt, rain, ROS, TF, and W. Dominant mechanism and mean W and SWE dates from seasonality analysis are also included.	
	[duration]_[mechanism] e.g., 24-h_W	**Data Dimension**: 207,173 (R) × 9 (C) **C1**: Latitude; **C2**: Longitude; **C3-C9:** IDF values with ARIs of 2, 5, 10, 25, 50, 100, and 500 years, in mm.
/IDF_curves_[Year]_detrend/e.g., /IDF_curves_CY_detrend/	IDF values and their 90% confidence intervals using detrended AM data with durations of 24 h, 48 h, and 72 h driven by different mechanisms from 1951-2013.	
	[duration]_[mechanism] e.g., 24-h_W	**Data Dimension**: 207,173 (R) × 9 (C) **C1**: Latitude; **C2**: Longitude; **C3-C9:** IDF values with ARIs of 2, 5, 10, 25, 50, 100, and 500 years, in mm.
/trend_results_[year] e.g., /trend_results_CY/	Sen’s slope of Mann-Kendall trend in AM series with different mechanisms in 1951-2013.	
	[duration]_[mechanism] e.g., 24-h_W	**Data Dimension**: 207,173 (R) × 3 (C) **C1**: Latitude; **C2**: Longitude; **C3**: Sen’s slope in mm/yr.

*Note: In “Data Description”, C = column, R = Row. C[i] indicates the ith column of a data file.

## Technical Validation

Due to the absence of data for direct NG-IDF curve evaluation, the evaluation focuses on W, which serves as the source data for deriving NG-IDF curves. Our specific focus was on evaluating the model’s simulated daily SWE, which is a key variable used in the W calculation besides precipitation data from the climate dataset. Currently, the NRCS SNOTEL network provides daily SWE measurements under open condition at approximately 800 stations in the western United States. To ensure quality, SNOTEL data was subject to a rigorous three-stage quality control filter^20^ and is subsequently corrected for snowfall undercatch^27^. The resulting data set, called bias-corrected quality-controlled (BCQC) SNOTEL data, can be accessed at https://climate.pnnl.gov/?category=Hydrology. Sun, *et al*.^25^ provided a detailed description of the comprehensive validation of DHSVM SWE simulation against SNOTEL data, and only a brief overview is presented here. Briefly, they selected 246 SNOTEL stations that shared the longest common period (2007–2013) of BCQC daily SWE records and evaluated the SWE simulation skill using three metrics: Nash-Sutcliffe Efficiency (NSE), bias in mean annual peak SWE (PEAK.ERR), and bias in the timing of peak SWE (PDATE.ERR). The results indicated that NSE of daily SWE was greater than 0.6 at 75% of the stations, absolute PEAK.ERR was less than or equal to 25% at 67% of the stations, and absolute PDATE.ERR was less than or equal to 14 days at 67% of the stations. These findings suggest that the calibrated DHSVM is capable of replicating the observed SWE dynamics at most of the stations using the regionalized snow parameters and can support large-domain hydrological applications. For more information, readers are directed to Sun, *et al*.^25^.

However, the data availability is rather limited for under-canopy SWE and most are short-term, discontinuous, point-scale measurements. Previous studies^27,28,67–71^ have extensively validated the ability of DHSVM to simulate snow and streamflow in various vegetated watersheds across the CONUS. For instance, in an extensive evaluation of 30 hydrological models, Beckers, *et al*.^69^ determined that DHSVM was the most suitable for hydrological modeling in forested environments. Du, *et al*.^67^ demonstrated that DHSVM effectively replicates the dynamics of snowpack, soil water content, and streamflow patterns in the forested Mica Creek Experimental Watershed in northern Idaho. Cristea, *et al*.^70^ confirmed that DHSVM accurately reproduces the dynamics of snow and streamflow in the forested Tuolumne basin of the Sierra Nevada, California. Sun, *et al*.^28^ further improved the DHSVM canopy model by incorporating canopy gap structure and verified its high accuracy in simulating snow under various canopy conditions, ranging from open to dense forest to canopy gaps, using field data from the University of Idaho Experimental Forest near Moscow, Idaho.

## Usage Notes

**Fig. 2:**
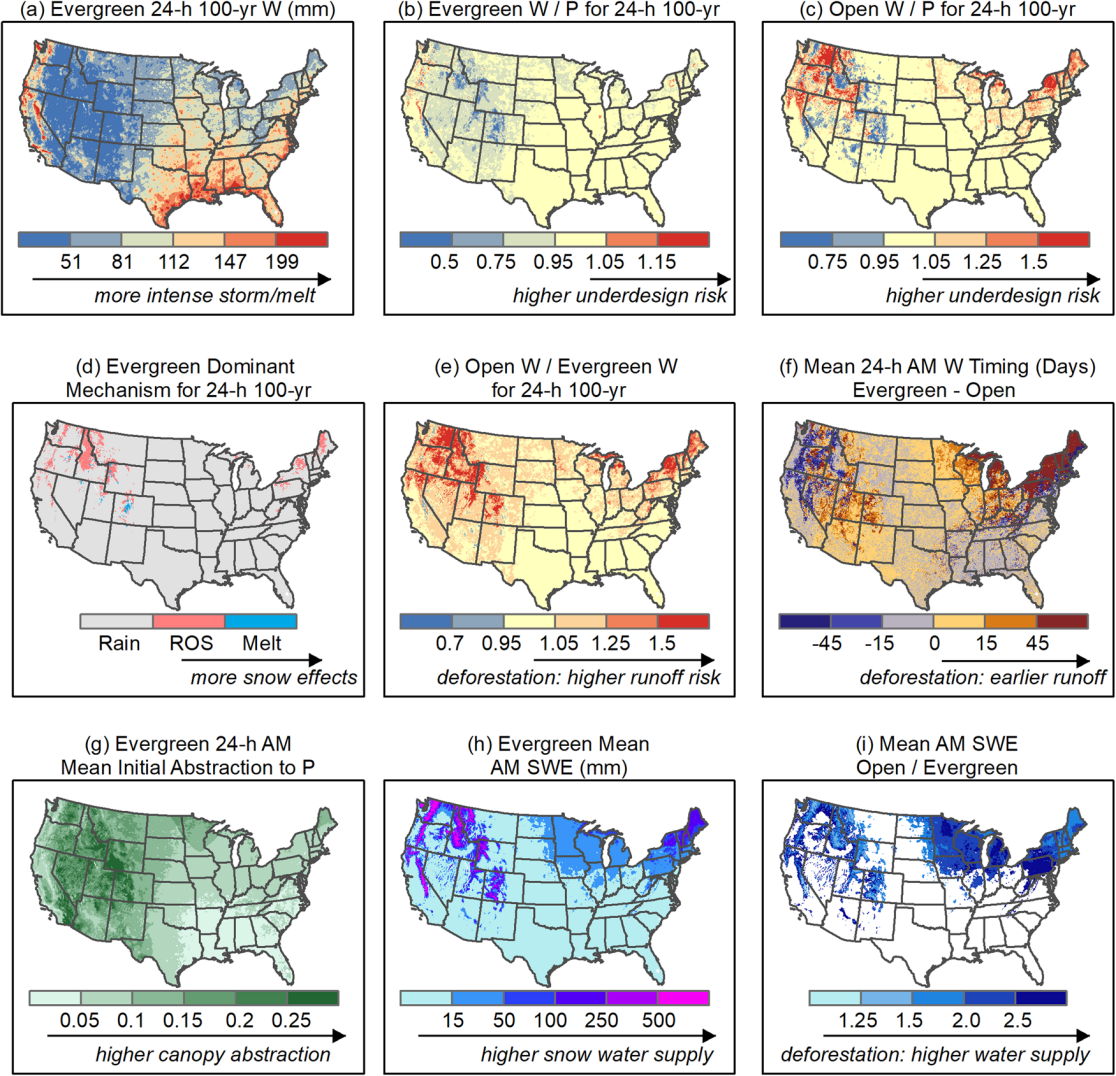
Example uses of NG-IDF datasets for nine LULCs, illustrated with calendar year AM data.

The NG-IDF 2.0 datasets provided in Table 2 are readily applicable for diverse hydrological applications across the CONUS without requiring further modifications. We present five data usage applications here as shown in Fig. 2, but our choices are not exhaustive.

1. *Assess design risk with the use of standard PREC-IDF curves*. To conduct hydrological design and analyses, users can obtain information on the magnitude of extreme W events and their associated P events for each LULC at any desired location (Fig. 2a,b,c).
2. *Plan various scenarios of LULC change and assess their effects on water supply and the risk of flooding*. Effective management of water resources requires careful planning for changes in LULC, which takes into account potential impact on both water supply and the risk of flooding. Here the NG-IDF 2.0 datasets that provide insights into the potential effects of LULC change on both snow water supply and flood risk are essential for effective LULC planning and sustainable water management (Fig. 2e,h,i).
3. *Offer physical insights into changes in runoff timing and mechanisms resulting from modifications in LULC*. The NG-IDF datasets offer runoff mechanisms (such as rain, snowmelt, and ROS) for each W event and seasonality for every LULC, allowing users to not only quantify changes in extreme W events but also comprehend the reasons for these changes (Fig. 2d,f). Moreover, the classification of the dominant runoff mechanism enables the development of mixed populations in the frequency of W events^72,73^.
4. *Enhance the standard hydrological design method by quantifying the spatial heterogeneity in LULC initial abstractions*. The TR-55, which is a commonly used IDF design method, employs a fixed ratio across the CONUS to represent canopy initial abstraction regardless of LULC variations. The NG-IDF 2.0 datasets can systematically investigate the canopy initial abstraction ratio for nine LULCs across the CONUS, providing an opportunity to enhance runoff prediction accuracy when using the standard IDF design method (Fig. 2g).
5. *Provide spatial runoff data for to support downstream modeling applications, such as flood inundation modeling*. In addition to integrating with rainfall-runoff models like TR-55 for assessing flood risk in hydrological design, the 6 km W datasets can be employed as inputs for hydrodynamic models like Rapid Infrastructure Flood Tool (RIFT)^74^ to enhance the accuracy of flood depth predictions (Fig. 2a), particularly for events triggered by snowmelt or ROS flooding, thus improving the existing Federal Emergency Management Agency (FEMA) Special Flood Hazard Area (SFHA) maps^75^.

Finally, it is important to acknowledge that the datasets are derived from a singular canopy condition, representing the average of a cluster. If the local canopy features vary significantly from this cluster average, the resulting information will differ. Moving forward, our intention is to create a cloud NG-IDF computing tool that allows users to input their specific local canopy attributes, enabling the generation of NG-IDF curves for any canopy condition related to each LUCL.

### Supplementary information

Supporting information

## Data Availability

The DHSVM source code is available at https://github.com/pnnl/DHSVM-PNNL Source codes that are used to develop and analyze the data are available at https://github.com/hydro-yan/NG-IDF The BCQC SNOTEL data are available at https://climate.pnnl.gov/?category=Hydrology.
