# Supplementary material for: Next-Generation Intensity-Duration-Frequency Curves for Diverse Land across the Continental United States
Source: Sci Data. 2023 Dec 4;10:863. doi: 10.1038/s41597-023-02680-4 (PMC10695926; doi:10.1038/s41597-023-02680-4)
Supplement: Supplementary file 1 — Supporting information [file 41597_2023_2680_MOESM1_ESM.docx]

Supporting Information for

**Next-Generation Intensity-Duration-Frequency Curves for Diverse Land across the Continental United States**

Hongxiang Yan^1*^; Zhuoran Duan^1,2^; Mark S. Wigmosta^1,2^; Ning Sun^1^; Ethan D. Gutmann^3^; Bert Kruyt^3^; and Jeffrey R. Arnold^4^

^1^ Earth Systems Science Division, Pacific Northwest National Laboratory, Richland, WA, USA

^2^ Department of Civil and Environmental Engineering, University of Washington, Seattle, WA, USA

^3^ National Center for Atmospheric Research, Boulder, CO, USA

^4^ MITRE Corporation, McLean, VA, USA

Corresponding author: Hongxiang Yan ([hongxiang.yan@pnnl.gov](mailto:hongxiang.yan@pnnl.gov))

**Table S1.** Cluster ground snow process parameters for each cluster over the CONUS.

| **Cluster** | **Ground Snow Parameters** | | | |
| --- | --- | --- | --- | --- |
|  | **Fresh Snow Albedo  (α_max)** | **Albedo Decay  Coefficient  during Snow  Accumulation (λ_A)** | **Albedo Decay  Coefficient  during Snowmelt (λ_M)** | **Temperature Threshold for Precipitation to be Completely Snowfall (T_s, °C)** |
| C1-Alpine | 0.85 | 0.93 | 0.90 | 3.3 |
| C2-Maritime | 0.84 | 0.93 | 0.89 | 2.4 |
| C3-Southern | 0.84 | 0.93 | 0.89 | 2.4 |
| C4-Northern | 0.85 | 0.98 | 0.87 | 1.3 |
| C5-Interior | 0.84 | 0.93 | 0.84 | 2.0 |

**Table S2.** Cluster canopy parameters of **evergreen forest** developed for the CONUS runs.

| **Canopy Parameter** | | **Cluster** | | | | |
| --- | --- | --- | --- | --- | --- | --- |
|  |  | **C1-Alpine** | **C2-Maritime** | **C3-Southern** | **C4-Northern** | **C5-Interior** |
| Overstory Max LAI | | 7.1 | 7.1 | 3.2 | 4.1 | 3.5 |
| Understory Max LAI | | 0.5 | 1.6 | 1.2 | 0.7 | 0.4 |
| Overstory Height (m) | | 16.1 | 24.3 | 16.2 | 15.2 | 12.6 |
| Understory Height (m) | | 0.5 | 0.9 | 0.7 | 0.5 | 0.4 |
|  |  |  |  |  |  |  |
| Monthly LAI Ratio to Max LAI | January | 1.00 | 1.00 | 1.00 | 1.00 | 1.00 |
|  | February | 1.00 | 1.00 | 1.00 | 1.00 | 1.00 |
|  | March | 1.00 | 1.00 | 1.00 | 1.00 | 1.00 |
|  | April | 1.00 | 1.00 | 1.00 | 1.00 | 1.00 |
|  | May | 1.00 | 1.00 | 1.00 | 1.00 | 1.00 |
|  | June | 1.00 | 1.00 | 1.00 | 1.00 | 1.00 |
|  | July | 1.00 | 1.00 | 1.00 | 1.00 | 1.00 |
|  | August | 1.00 | 1.00 | 1.00 | 1.00 | 1.00 |
|  | September | 1.00 | 1.00 | 1.00 | 1.00 | 1.00 |
|  | October | 1.00 | 1.00 | 1.00 | 1.00 | 1.00 |
|  | November | 1.00 | 1.00 | 1.00 | 1.00 | 1.00 |
|  | December | 1.00 | 1.00 | 1.00 | 1.00 | 1.00 |

**Table S3.** Cluster canopy parameters of **mixed forest** developed for the CONUS runs.

| **Canopy Parameter** | | **Cluster** | | | | |
| --- | --- | --- | --- | --- | --- | --- |
|  |  | **C1-Alpine** | **C2-Maritime** | **C3-Southern** | **C4-Northern** | **C5-Interior** |
| Overstory Max LAI | | 5.8 | 6.4 | 4.1 | 4.6 | 4.5 |
| Understory Max LAI | | 0.5 | 1.6 | 1.2 | 0.7 | 0.4 |
| Overstory Height (m) | | 15.0 | 21.0 | 18.6 | 17.5 | 19.5 |
| Understory Height (m) | | 0.5 | 0.9 | 0.7 | 0.5 | 0.4 |
|  |  |  |  |  |  |  |
| Monthly LAI Ratio to Max LAI | January | 0.27 | 0.48 | 0.34 | 0.27 | 0.33 |
|  | February | 0.30 | 0.42 | 0.43 | 0.31 | 0.39 |
|  | March | 0.34 | 0.63 | 0.53 | 0.40 | 0.50 |
|  | April | 0.48 | 0.78 | 0.74 | 0.49 | 0.75 |
|  | May | 0.72 | 1.00 | 0.99 | 0.73 | 0.94 |
|  | June | 1.00 | 0.96 | 1.00 | 0.94 | 1.00 |
|  | July | 0.96 | 0.95 | 0.98 | 1.00 | 0.99 |
|  | August | 0.84 | 0.93 | 0.95 | 0.91 | 0.99 |
|  | September | 0.74 | 0.85 | 0.84 | 0.84 | 0.98 |
|  | October | 0.44 | 0.69 | 0.66 | 0.52 | 0.75 |
|  | November | 0.37 | 0.46 | 0.41 | 0.35 | 0.42 |
|  | December | 0.23 | 0.41 | 0.32 | 0.28 | 0.34 |

**Table S4.** Cluster canopy parameters of **crop** developed for the CONUS runs.

| **Canopy Parameter** | | **Cluster** | | | | |
| --- | --- | --- | --- | --- | --- | --- |
|  |  | **C1-Alpine** | **C2-Maritime** | **C3-Southern** | **C4-Northern** | **C5-Interior** |
| Overstory Max LAI | |  |  |  |  |  |
| Understory Max LAI | | 4.0 | 4.0 | 4.0 | 4.0 | 4.0 |
| Overstory Height (m) | |  |  |  |  |  |
| Understory Height (m) | | 1.7 | 1.7 | 1.7 | 1.7 | 1.7 |
|  |  |  |  |  |  |  |
| Monthly LAI Ratio to Max LAI | January | 0.04 | 0.25 | 0.33 | 0.10 | 0.29 |
|  | February | 0.06 | 0.29 | 0.40 | 0.10 | 0.34 |
|  | March | 0.08 | 0.43 | 0.51 | 0.14 | 0.54 |
|  | April | 0.13 | 0.58 | 0.61 | 0.22 | 0.79 |
|  | May | 0.34 | 0.62 | 0.66 | 0.37 | 0.87 |
|  | June | 0.81 | 0.75 | 0.86 | 0.72 | 0.85 |
|  | July | 1.00 | 1.00 | 1.00 | 1.00 | 1.00 |
|  | August | 0.85 | 0.95 | 0.92 | 0.94 | 0.94 |
|  | September | 0.39 | 0.60 | 0.68 | 0.45 | 0.63 |
|  | October | 0.16 | 0.30 | 0.43 | 0.19 | 0.37 |
|  | November | 0.11 | 0.27 | 0.33 | 0.15 | 0.37 |
|  | December | 0.06 | 0.26 | 0.30 | 0.11 | 0.28 |

**Table S5.** Cluster canopy parameters of **grass** developed for the CONUS runs.

| **Canopy Parameter** | | **Cluster** | | | | |
| --- | --- | --- | --- | --- | --- | --- |
|  |  | **C1-Alpine** | **C2-Maritime** | **C3-Southern** | **C4-Northern** | **C5-Interior** |
| Overstory Max LAI | |  |  |  |  |  |
| Understory Max LAI | | 2.5 | 2.5 | 2.5 | 2.5 | 2.5 |
| Overstory Height (m) | |  |  |  |  |  |
| Understory Height (m) | | 0.3 | 0.3 | 0.4 | 0.3 | 0.3 |
|  |  |  |  |  |  |  |
| Monthly LAI Ratio to Max LAI | January | 0.44 | 0.48 | 0.47 | 0.18 | 0.29 |
|  | February | 0.34 | 0.56 | 0.61 | 0.20 | 0.34 |
|  | March | 0.35 | 0.73 | 0.79 | 0.27 | 0.51 |
|  | April | 0.60 | 0.85 | 0.92 | 0.47 | 0.77 |
|  | May | 0.92 | 0.96 | 1.00 | 0.92 | 1.00 |
|  | June | 1.00 | 1.00 | 0.96 | 1.00 | 0.89 |
|  | July | 0.90 | 0.95 | 0.84 | 0.91 | 0.80 |
|  | August | 0.71 | 0.85 | 0.77 | 0.79 | 0.73 |
|  | September | 0.54 | 0.74 | 0.78 | 0.55 | 0.60 |
|  | October | 0.39 | 0.58 | 0.59 | 0.33 | 0.40 |
|  | November | 0.37 | 0.51 | 0.46 | 0.25 | 0.36 |
|  | December | 0.21 | 0.52 | 0.40 | 0.21 | 0.29 |

**Table S6.** Cluster canopy parameters of **pasture** developed for the CONUS runs.

| **Canopy Parameter** | | **Cluster** | | | | |
| --- | --- | --- | --- | --- | --- | --- |
|  |  | **C1-Alpine** | **C2-Maritime** | **C3-Southern** | **C4-Northern** | **C5-Interior** |
| Overstory Max LAI | |  |  |  |  |  |
| Understory Max LAI | | 2.5 | 2.5 | 2.5 | 2.5 | 2.5 |
| Overstory Height (m) | |  |  |  |  |  |
| Understory Height (m) | | 0.3 | 0.3 | 0.4 | 0.3 | 0.3 |
|  |  |  |  |  |  |  |
| Monthly LAI Ratio to Max LAI | January | 0.11 | 0.36 | 0.32 | 0.13 | 0.21 |
|  | February | 0.12 | 0.39 | 0.38 | 0.13 | 0.25 |
|  | March | 0.16 | 0.64 | 0.59 | 0.26 | 0.41 |
|  | April | 0.30 | 0.93 | 0.85 | 0.49 | 0.73 |
|  | May | 0.64 | 1.00 | 0.96 | 0.84 | 0.97 |
|  | June | 1.00 | 0.82 | 1.00 | 0.92 | 0.97 |
|  | July | 0.91 | 0.86 | 0.91 | 0.99 | 1.00 |
|  | August | 0.69 | 0.77 | 0.89 | 1.00 | 0.96 |
|  | September | 0.54 | 0.57 | 0.81 | 0.75 | 0.71 |
|  | October | 0.32 | 0.47 | 0.52 | 0.43 | 0.48 |
|  | November | 0.21 | 0.41 | 0.35 | 0.29 | 0.33 |
|  | December | 0.14 | 0.37 | 0.25 | 0.20 | 0.22 |

**Table S7.** Cluster canopy parameters of **shrub** developed for the CONUS runs.

| **Canopy Parameter** | | **Cluster** | | | | |
| --- | --- | --- | --- | --- | --- | --- |
|  |  | **C1-Alpine** | **C2-Maritime** | **C3-Southern** | **C4-Northern** | **C5-Interior** |
| Overstory Max LAI | |  |  |  |  |  |
| Understory Max LAI | | 2.3 | 2.3 | 2.3 | 2.3 | 2.3 |
| Overstory Height (m) | |  |  |  |  |  |
| Understory Height (m) | | 0.7 | 1.5 | 1.0 | 0.7 | 0.6 |
|  |  |  |  |  |  |  |
| Monthly LAI Ratio to Max LAI | January | 0.19 | 0.50 | 0.50 | 0.24 | 0.45 |
|  | February | 0.21 | 0.56 | 0.60 | 0.26 | 0.52 |
|  | March | 0.30 | 0.76 | 0.79 | 0.33 | 0.63 |
|  | April | 0.48 | 0.95 | 0.96 | 0.51 | 0.84 |
|  | May | 0.85 | 0.98 | 1.00 | 0.96 | 1.00 |
|  | June | 1.00 | 1.00 | 0.97 | 1.00 | 0.88 |
|  | July | 0.83 | 0.96 | 0.82 | 0.77 | 0.77 |
|  | August | 0.66 | 0.93 | 0.91 | 0.61 | 0.77 |
|  | September | 0.50 | 0.80 | 1.00 | 0.51 | 0.69 |
|  | October | 0.37 | 0.65 | 0.75 | 0.40 | 0.55 |
|  | November | 0.35 | 0.56 | 0.52 | 0.35 | 0.48 |
|  | December | 0.21 | 0.58 | 0.48 | 0.20 | 0.48 |

**Table S8.** Cluster canopy parameters of **wetland** developed for the CONUS runs.

| **Canopy Parameter** | | **Cluster** | | | | |
| --- | --- | --- | --- | --- | --- | --- |
|  |  | **C1-Alpine** | **C2-Maritime** | **C3-Southern** | **C4-Northern** | **C5-Interior** |
| Overstory Max LAI | | 4.0 | 4.0 | 4.0 | 4.0 | 4.0 |
| Understory Max LAI | | 2.5 | 2.5 | 2.5 | 2.5 | 2.5 |
| Overstory Height (m) | | 9.6 | 6.6 | 3.6 | 2.6 | 1.8 |
| Understory Height (m) | | 0.5 | 0.9 | 0.7 | 0.5 | 0.4 |
|  |  |  |  |  |  |  |
| Monthly LAI Ratio to Max LAI | January | 0.16 | 0.48 | 0.51 | 0.32 | 0.26 |
|  | February | 0.32 | 0.56 | 0.62 | 0.45 | 0.27 |
|  | March | 0.24 | 0.73 | 0.74 | 0.34 | 0.36 |
|  | April | 0.33 | 0.85 | 0.90 | 0.45 | 0.61 |
|  | May | 0.62 | 0.96 | 0.95 | 0.68 | 0.87 |
|  | June | 0.91 | 1.00 | 1.00 | 0.97 | 1.00 |
|  | July | 1.00 | 0.95 | 1.00 | 1.00 | 0.90 |
|  | August | 0.92 | 0.85 | 0.98 | 0.95 | 0.71 |
|  | September | 0.69 | 0.74 | 0.97 | 0.81 | 0.54 |
|  | October | 0.27 | 0.58 | 0.85 | 0.42 | 0.39 |
|  | November | 0.24 | 0.51 | 0.63 | 0.30 | 0.30 |
|  | December | 0.15 | 0.52 | 0.51 | 0.26 | 0.27 |
